# Evaluation of the Mechanical and Adhesion Characteristics of Indirect Restorations Manufactured with Three-Dimensional Printing

**DOI:** 10.3390/polym16050613

**Published:** 2024-02-23

**Authors:** Cem Peskersoy, Aybeniz Oguzhan

**Affiliations:** Faculty of Dentistry, Ege University, 35030 Izmir, Turkey; aybeniz.oguzhan@ege.edu.tr

**Keywords:** 3D printing, additive manufacturing, subtractive manufacturing, marginal fit, bond strength

## Abstract

The aim of this study was to investigate the marginal fit and bond strength characteristics of onlay restorations manufactured by three-dimensional printing (Varseo XS, Bego GmbH, Bremen, Germany) and CAD/CAM (CAMcube, Montreal, QC, Canada) systems. Class II onlay cavities on sixty mandibular molars were prepared in cavities and restored in three separate groups using different fabrication methods. Digital and conventional impressions were taken to design the restorations in the CAD system (DWOS, Straumann GmbH, Freiburg, Germany). To evaluate the marginal fit and void volumes, all specimens were scanned with microcomputed tomography. A microshear test was performed to compare the bond strength of the restorations to the tooth surface. The marginal fit values measured for the 3D-printed and CAD/CAM onlay restorations were found to be at clinically acceptable levels (<120 µm), and no significant difference could be observed between the three different fabrication methods (*p* > 0.05). According to the microshear test results, the CAD/CAM group had the highest bond strength values before (34.82 MPa) and after (26.87 MPa) thermal cycling (*p* < 0.05), while the 3D-printed and conventionally produced onlays had similar results (*p* < 0.05). 3D printing technology is a promising option for indirect restorations; however, the post-production phase is as crucial as the printing and cementation phases.

## 1. Introduction

The success of restorative treatments depends on the strength of the restoration, marginal fit, and its bond strength to the tooth structures [1]. To achieve better long-term clinical performance and success in these treatments, new digital techniques and materials have emerged [1,2]. Due to advancements in technology and industry, treatment methods and devices have evolved from subtractive or conventional methods to more complex additive manufacturing [3]. The emergence and popularity of computer-aided applications in dental practice have given rise to the concept of digital dentistry [2].

In the workflow of digital dentistry, computer-aided applications allow the final product to be created using two different manufacturing techniques. These techniques include subtractive manufacturing and additive manufacturing. Subtractive manufacturing is a method in which the final product is created from a material block through milling guided by a computer numerical control (CNC) device. The aim of this process is to obtain a specific three-dimensional geometry. The design data are arranged according to the milling tool settings on the CNC device [3]. Conversely, the fundamental principle of additive manufacturing involves the layer-by-layer assembly of a monomeric material through a computer-controlled energy source, resulting in the production of complex three-dimensional shapes [3,4].

The subtractive method for producing restorations may lead to defects, such as notches and microcracks, at the marginal edges and surfaces due to milling processes [5]. Another significant limitation of subtractive manufacturing is the generation of excess leftover material to obtain the final restoration. Strub et al. reported that approximately 90% of the material was wasted in their studies employing subtractive manufacturing. The inability to reuse excess material leads to environmental and economic concerns, such as waste management, energy consumption, challenges in designing, and material selection. [6]. In contrast, additive manufacturing produces only the necessary parts and allows for the reuse of the polymer resin required for printing, avoiding the generation of leftover material. In addition, taking some savings measures, such as producing hollow models or reducing the number of supports, are some of the advantages of the additive method. These aspects make additive manufacturing a highly economical process [7,8]. In contemporary times, additive manufacturing methods have gained preference as a technique for overcoming the limitations associated with subtractive manufacturing. The terms “3D printing” and “rapid prototyping technology” are employed to delineate devices that utilize additive manufacturing methods. Since additive manufacturing is an economically viable process, the incorporation of 3D printing systems and compatible resins into dental production has rendered these technologies a substantial focal point of interest.

In 3D printing, a layer is incrementally produced from a polymer material in powder or liquid form until the desired object shape is achieved [9]. The 3D printing of biomedical polymers, which involves the utilization of fillers and biocompatible polymers, has potential for adjusting physicochemical, mechanical, and biological properties. To this end, layer manufacturing technologies operating with photopolymerizable resins, such as stereolithography (SLA) and digital laser projection (DLA), are crucial for providing precise, versatile, and controlled manipulation [10]. Besides the viscosity, the ingredients, filler ratio, and chemical compounds in the polymer, such as initiators, accelerators, etc., have a direct effect on the final product. Therefore, to date, various resin systems have been designed, produced, and developed for 3D printing in dentistry. VarseoSmile Crown Plus (Bego, Bremen, Germany) was the first resin system specifically designed for permanent restorations manufactured in the correct tooth color. This restoration is a ceramic-infiltrated hybrid composite resin composed of a methacrylic acid ester matrix, and it can be classified as a resin-matrix ceramic [11].

Resin-matrix ceramics combine the positive properties of all-ceramic materials and composite materials [12]. The stiffness and rigidity of the ceramic is combined with the elasticity of the resin ingredient [13]. The color stability of the ceramic resin prevents the material from discolorizing depending on time via liquid absorption in vivo. Both composite resins and ceramics have proven their success in indirect restoration applications for many years by long-term follow-up studies [13,14,15]. However, both materials exhibit some adverse characteristics in terms of the long-term success of restorations. Resin-based composites are complex materials created through the mechanism of free radical polymerization. Polymerization shrinkage in resin composites generates stress at the interface between the composite restoration and the tooth substance [16]. However, ceramics, despite their high durability, are inherently brittle materials and sensitive. Additionally, ceramics have an abrasive effect on opposite teeth [3]. Therefore, producing a resin matrix ceramic consisting of a matrix reinforced with nano or nano-hybrid ceramic fillers had been thought to be a challenge to overcome and to become an alternative to traditional ceramics and resin composites [2,3,8].

To achieve precise, compatible, and superior mechanical properties in restorations through 3D printing and to establish the optimal parameters for routine clinical application, there is a pressing need for advanced investigations in this domain. Certain pivotal factors must be thoroughly scrutinized before the integration of a new material and manufacturing method into regular dental practices. Specific parameters, such as functional and aesthetic characteristics, marginal fit, bond strength, mechanical durability, and resistance to thermal stress, hold critical significance for ensuring optimal clinical performance and the long-term success of restorations. The primary objective of our study was to compare the additive manufacturing, subtractive manufacturing, and traditional manufacturing methods in dental restoration production in terms of marginal fit and bond strength. The null hypothesis of our study was that there would be no difference between the three different restoration production techniques tested in terms of marginal fit and bond strength.

## 2. Materials and Methods

### 2.1. Examination of Marginal Fit

Marginal adaptation of the restorations was compared using the microcomputed tomography method, which is considered a non-invasive and objective technique, to observe the specimens and measure the micron-scale incompatibilities.

#### 2.1.1. Cavity Preparation

The freshly extracted caries-free first mandibular molars (*n* = 60) were stored in a solution saturated with distilled water. The teeth were extracted for periodontal orthodontic reasons. Then, each tooth was embedded in acrylic resin (Duracryl Self Cure, Duradent Erk Dental, Izmir, Turkey) within cylindrical plastic blocks, measuring 1.6 cm in diameter and 2 cm in length, with the enamel–cement junction at the surface.

**Figure 1:**
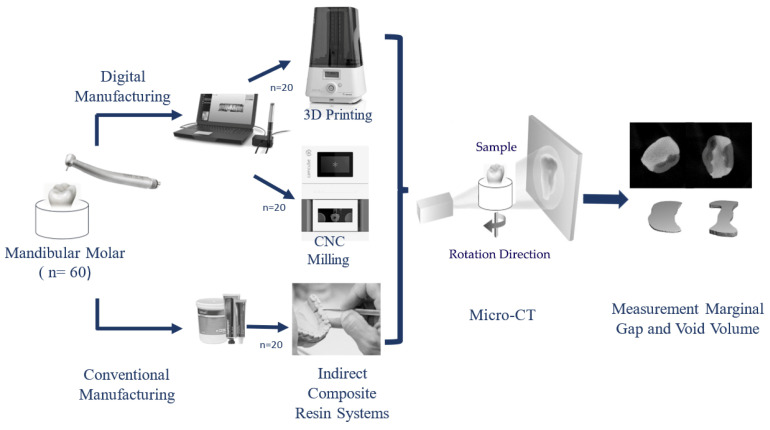
Research procedure diagram.

Standardized occlusal preparations were performed on all 60 teeth by the same dentist. Cavities were prepared using preparation burs (DIMEI Piranha; Dimei Dental Material Co., Anyang, Henan Province, China) with cooling provided by an air–water syringe (KaVo S608C Smart, KaVo Dental GmbH, Biberach, Germany). The aim of the preparation process was to mimic substance loss conditions in teeth, setting the cavity depth to 3 mm between the occlusal and pulpal walls and the occlusal cavity width to one-third of the distance between the tubercles. In Class II (MOD) onlay preparation, the margin between the distal and mesial gingival wall was positioned 1–1.5 mm above the enamel–cement junction. The prepared proximal wall had a mesiodistal width of 2 mm and a buccolingual width of 3 mm. The axial wall was 1.5 mm in length occluso-gingivally. The nonfunctional lingual tubercles were reduced to a minimum of 1.5 mm. The internal angles of the cavity were rounded to eliminate sharp corners and undercuts. Depth and width measurements of the cavities were obtained using a periodontal probe (Falcon Periodontal Williams Probe, Lucca, Italy). The 60 teeth prepared for onlay restoration were randomly divided into three groups, each containing 20 teeth, for further restoration: 3D (3D printing), CS (subtractive manufacturing), and SG (indirect composite resin) (Figure 1).

#### 2.1.2. Impression

For the groups in which computer-aided design and manufacturing through additive and subtractive methods were conducted, digital intraoral scans of the teeth were obtained using a digital intraoral scanner (Virtuo Vivo TM Straumann, Dental Wings, Inc., Montreal, QC, Canada). The three-dimensional digital images of the scanned samples were exported and saved in standard tessellation language (STL) and polygon file format (PLY) files. For the group that was restored on stone models with an indirect composite resin system, impressions of the prepared teeth were obtained using conventional methods (Optosil Comfort Putty and Xantopren L Blue, Heraeus Kulzer, Hanau, Germany). Subsequently, stone models were created.

#### 2.1.3. Restoration Design

The samples designed in the CS group were analyzed using Sum3D MillBox software (version 2020, CIM System, Milano, Italy), while the restorations in the 3D group were analyzed using Exocad software 3.0 (Exocad Gmbh, Darmstadt, Germany).

#### 2.1.4. Manufacturing of Onlay Restorations

Since the Virtuo Vivo software (DWOS 9, version 3.0) lacked a milling system, it was utilized only during the impression stage. The design and production stages were carried out using different systems.

After completely designing the onlay restorations through computer software, the data in STL file format were transferred to the Bego Varseo XS device (Bego, Bremen, Germany) via USB. In this group, 20 samples were produced using digital light processing (DLP) technology with a 405 nm wavelength, 50 µm resolution (X, Y, and Z), and 0.25 mm/min printing speed using Varseo Smile Crown Plus (Bego, Bremen, Germany) permanent restoration resin on a 3D printing device. To minimize procedural variability, all samples were produced simultaneously at a constant room temperature of 23 °C. The printer was calibrated beforehand according to the manufacturer’s recommendations. After completing the printing process, the restorations were carefully removed from the printing platform using a spatula. The obtained samples were cleaned in an ultrasonic bath (Foshan Adelson Medical Devices Co., Foshan, China) for 480 s by adding a 96% ethanol solution, followed by gentle drying. For post-curing, the restorations were placed in the Bego Otoflash unit (Bego, Bremen, Germany). The samples were subjected to 1500 flashes at 10 Hz in an atmosphere containing nitrogen gas (1.0–1.2 bar). The restorations were then flipped and the post-curing process was repeated with 1500 auto flashes on the other side of the platform.

Onlay restorations produced from Cerasmart (GC Corp, Tokyo, Japan) resin nanoceramic blocks were cut out using a CAMcube M10 milling device (CAMcube, Montreal, QC, Canada). The parameters of the system were set as follows: 0.1 mm plunging, 20,000 RPM speed, 2000 feed-rate, and 3 mm roughing. To create a barrier facilitating easy separation, Imiseal (Imıcryl, Konya, Turkey) coating solution was applied to the inner and outer walls of the cavity stone models using a bond brush. A piece of Signum (Heraeus Kulzer, Hanau, Germany) indirect composite resin was placed in the cavity in a layered manner, adapting to the natural morphology, at a thickness of 1.5–2 mm. A light device (Woodpecker Led C, Guilin, China) was used for polymerization after each layer. After conducting the layering and morphology processing steps for the indirect composite resin, the prepared restorations were placed in a Bego Otoflash (Bego, Bremen, Germany) device for post-curing. The restorations underwent 1500 × 2 flashes with a nitrogen gas atmosphere at a frequency of 10 Hz.

#### 2.1.5. Cementation

##### Preparation of the Restoration Adherent Surface

The adherent surfaces of the onlays were continuously air-abraded for 10 s using alumina powder with an average particle size of 30 μm (Toye Corp., Shenzhen, China) at 2.5 bar. Then, the samples were ultrasonically cleaned for 6 min in 96% ethanol. After the ultrasonic bath, the restorations were dried, after which they were prepared for cementation. The silane agent Monobond (Ivoclar Vivadent, Schaan, Liechtenstein) was applied to the adherent surface of the restoration with a bond brush, and it was kept on the surface for 60 s. Subsequently, the adherent surface of the restoration was air-dried for 5 s.

##### Preparation of the Tooth Cavity Surface

**Figure 2:**
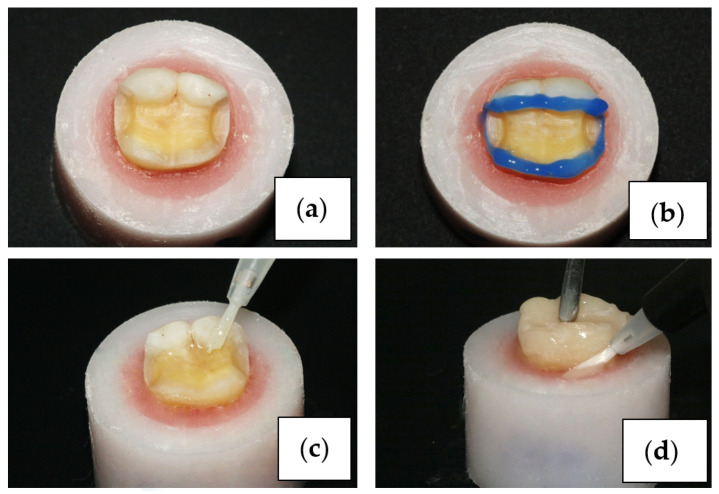
Cavity preparation (**a**), acid etching (**b**), adhesive application (**c**), cementation (**d**).

The enamel edges of the cavity were selectively etched with 37% orthophosphoric acid (Proetch, Promida, Eskisehir, Turkey), and the cavity was acidified for 30 s. Subsequently, the acid mixture was rinsed and air-dried (Figure 2). The Heliobond (Ivoclar Vivadent, Schaan, Liechtenstein) bonding agent was applied, and a 20 s waiting period was used for observation and air-thinning. Polymerization was carried out for 20 s with a light device (Woodpecker Led C, China).

After completing the preparation of the cavity and restoration surfaces, cementation was performed with RelyX U200 (3M ESPE, St. Paul, MN, USA) self-adhesive cement (Figure 2). Excess cement was removed with a brush, and light was applied for 40 s to each of the occlusal, mesial, distal, and lingual surfaces, for a total of 160 s of light exposure, to ensure polymerization. Polishing discs (Tor, Moscow, Russia) and polishing rubber tips were used to remove polymerized cement residues.

#### 2.1.6. Imaging with Micro-CT

Microcomputed tomography scanning of the teeth in the three groups was performed with a SCANCO Medical µCT 50 (SCANCO Medical, Brüttisellen, Switzerland). The device was operated with a scanning slice thickness of 20 μm, a voltage of 90 kV, a current of 155 μm, and an image integration speed of 300 ms. Each tooth was imaged for 15 min, and an average of 450 sections were obtained for each tooth.

##### Measurement of the Marginal Gap

Sections of all specimens were divided into three regions: the occlusal, mid-triple, and cervical regions. Within each region, 3 sections were selected for examination. To calculate the millimetric distance corresponding to the pixel length in Photoshop (version 2023, Adobe, San Jose, CA, USA), the inner diameter of the scanning tube (its exact length could be observed in the image) was used as a reference. The inner diameter of the scanning tube was measured in pixels (2305 pixels). In the logical length section, the actual known length of the scanning tube, in mm (47 mm), was entered, and the pixel equivalents were calculated in millimeters. The gaps between the tooth and the restoration on the examined sections were measured with a mm scale ruler under magnification in accordance with the measurement scale created. The measured values were recorded in MS Excel (Microsoft, Redmond, WA, USA).

##### Measurement of Void Volume in the Restoration Material

The voids in the restoration material were analyzed with the µCT Evaluation Program V6.5 (SCANCO Medical, Brüttisellen, Switzerland). The occlusal, middle triangular, and cervical regions of the teeth were measured for volumetric voids. In each section between the first and last sections representing these regions, restoration borders were drawn, and the voids in the material were measured. With the analysis software, all the measured sections were superimposed, and a three-dimensional analysis model was created.

The bone volume/total volume (BV/TV) value could be used to define the void volume per unit volume in the material in the selected region of analysis. This parameter was used to determine the level of porosity in this study. The higher the BV/TV ratio, the denser the material. The lower the BV/TV ratio, the greater the porosity. The BV/TV ratio was between 0 and 1. Micro-CT has been recognized as the gold-standard method for assessing porous microstructures [17]. The total volume–bone volume (TV-BV) was calculated as the void space in the material in mm^3^ by subtracting the nonporous volume from the total volume.

### 2.2. Bond Strength Analysis

In our study, a microshear test was performed to investigate the bond strengths of different restoration methods on dental tissues.

#### 2.2.1. Sample Preparation

**Figure 3:**
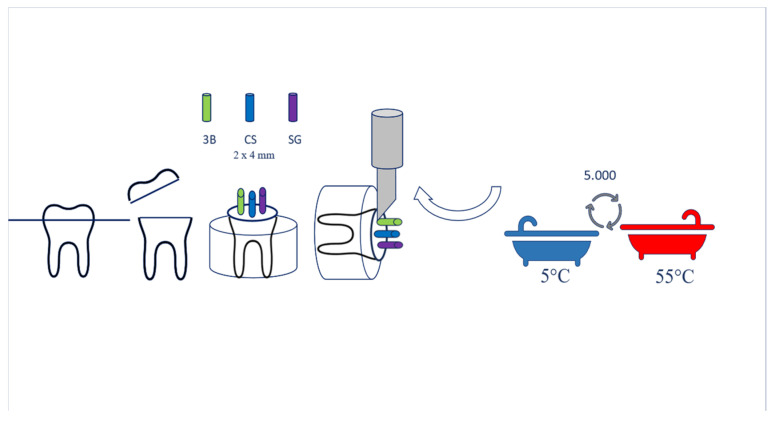
Microshear test and thermal cycling.

For the three study groups (3D, CS, and SG), a total of twelve caries-free and crack/fracture-free human molars were utilized. All teeth were horizontally sectioned from the coronal 1/3 region using an Isomet blade and machine (Buehler, Lake Bluff, IL, USA), perpendicular to the tooth axis (vertical plane), resulting in tooth sections that contained healthy dentin surfaces. To create a stable base, the teeth were embedded in plastic cylinder molds (1.6 × 2 cm) filled with acrylic resin (Duracryl Self Cure, Duradent Erk Dental, Izmir, Turkey) to a depth of 1 mm below the enamel–cement border. Subsequently, the dentin samples were polished under water, employing 600, 800, and 1200 grit sandpaper to achieve flat and smooth surfaces. Cylinder blocks with diameters of 2 mm and lengths of 4 mm were then prepared from Signum, Cerasmart, and VarseoSmile Crown Plus materials. Cylinder blocks composed of the three different materials were cemented to the dentin surface of the same tooth. While preparing both the models and the tooth surfaces for cementation, the materials used in the onlay cementation steps were applied in the same order (Figure 3).

#### 2.2.2. Microshear Bond Strength Test

Microshear bonding tests were conducted with a universal testing machine (Shimadzu AG-X, Tokyo, Japan). A knife-edged shear bar with a crosshead speed of 0.5 mm/min was employed at the bond interface (refer to Figure). The maximum shear load at the point of damage was meticulously recorded. The bond strength was subsequently calculated using the load at the point of damage (F) and the adhesive area (A): σ = F/A (N/mm^2^). The obtained microshear bond strength (mSBS) values were documented in units of MPa (Figure 3).

#### 2.2.3. Analysis of Fracture Types

Following the bond strength test, all the samples were thoroughly examined under a light microscope (Leica, V.4.2.0, Heerbrugg, Switzerland) at 2.0× magnification. The fracture types were carefully identified and recorded.

#### 2.2.4. Thermal Cycling (TC) Procedure

A total of 5000 thermal cycles were performed, alternating between 5 and 55 °C. This cumulative exposure to 5000 cycles took approximately 6 months in an oral environment [18]. Each temperature transition featured a dwell time of 20 s, with a 10 s transition period between temperature baths. After thermal cycling, the shear bond strength test was reiterated using identical procedures (Figure 3).

### 2.3. Statistical Analysis

To assess the collected data, the disparities among the mean values of the three groups were scrutinized via one-way analysis of variance (ANOVA). Post hoc analysis was conducted using Tukey’s multiple comparison test to discern the specific groups contributing to the observed differences. Tukey’s test and *t*-tests were employed to investigate distinctions in bond strength measurements. Furthermore, the chi-square test of independence was utilized to explore potential associations between production methods and fracture types. All the statistical analyses were performed with a significance level of 95% (*p* < 0.05).

## 3. Results

### 3.1. Evaluation of Marginal Fits in Manufacturing Methods

No statistically significant difference was found in the marginal fits of the 3D group and the SG group or in those of the 3D group and the CS group (*p* > 0.05). However, a statistically significant difference was observed in the mean marginal gap values of the SG group and the CS group (*p* < 0.05). The mean marginal gap values of all groups were within the clinically acceptable range (<120 µm) (Table 1). Upon analysis, it was determined that the group with the best mean marginal fit was the CS group, followed by the 3D group. However, the SG group exhibited weaker mean marginal fit values than the CS group (*p* < 0.05).

### 3.2. Evaluation of the Marginal Fits of Manufacturing Methods by Region

**Figure 4:**
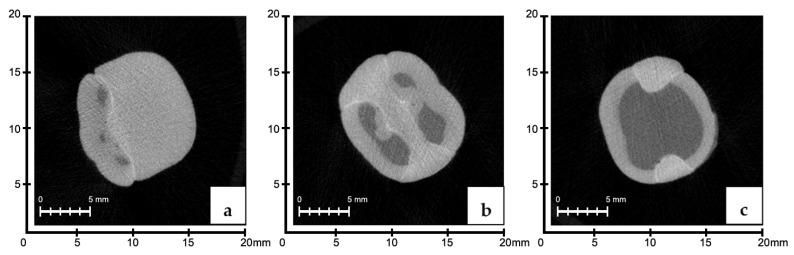
Occlusal (**a**), middle third (**b**), and cervical (**c**) regions of 3D group; micro-CT sections were measured.

**Figure 5:**
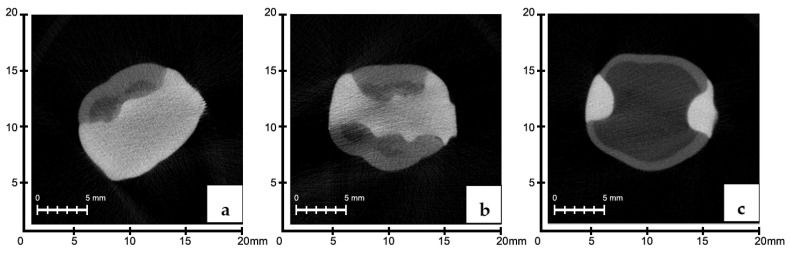
Occlusal (**a**), middle third (**b**), and cervical (**c**) regions of CS group; micro-CT sections were measured.

**Figure 6:**
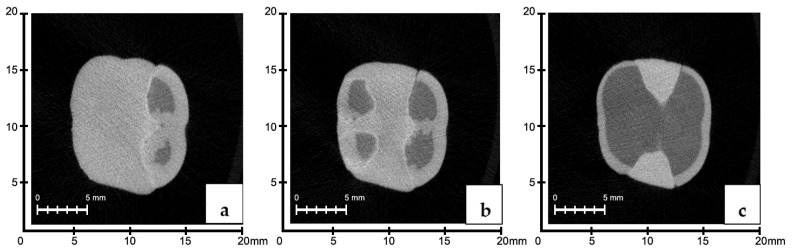
Occlusal (**a**), middle third (**b**), and cervical (**c**) regions of SG group; micro-CT sections were measured.

When the marginal range averages in the cervical region were evaluated, there were no significant differences among the groups (*p* > 0.05). In the middle third region, the SG group had greater mean marginal gap values than the CS and 3D groups (*p* < 0.05). The group with the highest marginal gap values in the occlusal region was determined to be the 3D group (*p* < 0.05) (Figure 4, Figure 5 and Figure 6).

### 3.3. Evaluation of Void Volumes in the Internal Structure of the Restoration According to Manufacturing Methods

**Figure 7:**
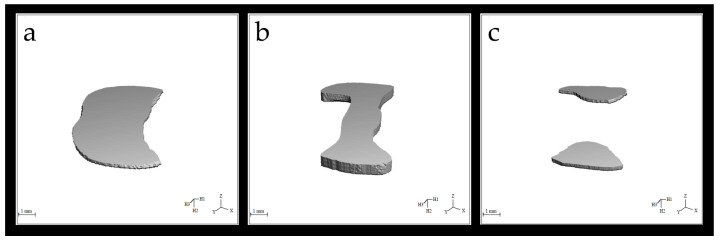
(**a**) Occlusal, (**b**) middle third, and (**c**) cervical 3D analysis model of the cavity volume in the internal structure of the restoration.

**Figure 8:**
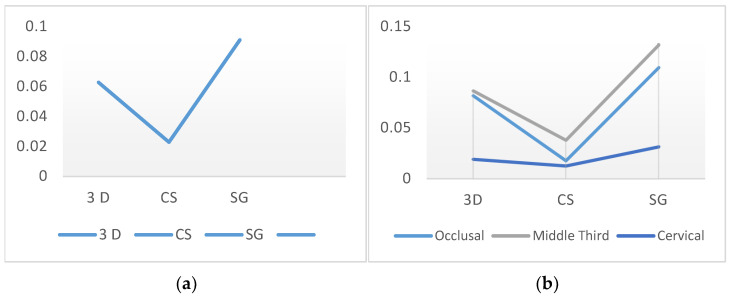
Measurement of void volume; (**a**) evaluation of void volumes in the internal structure of the restoration according to manufacturing methods, (**b**) evaluation of void volume (mm^3^) in the restoration internal structure according to region.

There were no significant differences in the mean cavity volumes among the 3D, CS, and SG groups (*p* > 0.05). The average void volume measured in the material in the restorations in the CS group was lower than that in the SG group (*p* ˂ 0.05) (Figure 7 and Figure 8).

### 3.4. Evaluation of Void Volume (mm^3^) in the Restoration Internal Structure According to Region

No significant difference was observed between the methods used for the middle third or cervical regions (*p* > 0.05). In the occlusal region, there were no significant differences in the void volumes of the 3D and SG groups (*p* > 0.05). However, in the occlusal region, the CS group exhibited significantly lower mean void volume values than the 3D and SG groups (*p* < 0.05) (Figure 7 and Figure 8).

### 3.5. Evaluation of the BV/TV Ratios According to the Production Method and Region

**Figure 9:**
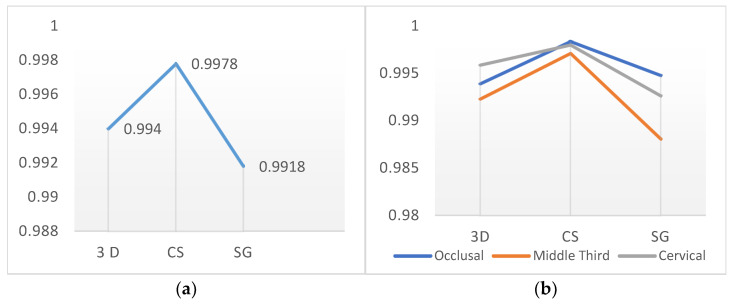
Evaluation of the BV/TV ratios according to the production method (**a**) and region (**b**).

When evaluating the BV/TV ratios, the CS group demonstrated significantly lower porosity within the material than the 3D and SG groups (*p* < 0.05). No significant differences were found in the mean BV/TV ratios of the 3D and SG groups (*p* > 0.05). The SG group exhibited the most porous and largest number of void structures among the specimens (*p* < 0.05). There were no significant differences in the 3D and SG groups in the middle third and cervical regions (*p* > 0.05). The 3D group exhibited a greater porosity rate in occlusal sections than the CS group (*p* < 0.05). In the middle third and cervical regions, the SG group had a greater void-to-volume ratio within the restored internal structure than the CS group (*p* < 0.05) (Figure 9).

### 3.6. Evaluation of Bond Strength Test Results

For all groups, a comparative analysis was conducted on the average void volume and bond strength, revealing a significant positive correlation between the variables (*p* < 0.05). Although the bond strength of the SG group increased after thermal cycling, this difference was not significant (*p* > 0.05). The CS group exhibited the highest mean bond strengths before and after thermal cycling (Table 2). However, a statistically significant decrease in bond strength was observed after thermal cycling (*p* < 0.05). Before and after thermal cycling, the CS group demonstrated superior bond strength compared with the 3D and SG groups (*p* < 0.05). There were no significant differences in the bond strengths of the 3D and SG groups before and after thermal cycling (*p* > 0.05).

### 3.7. Evaluation of Fracture Types

**Figure 10:**
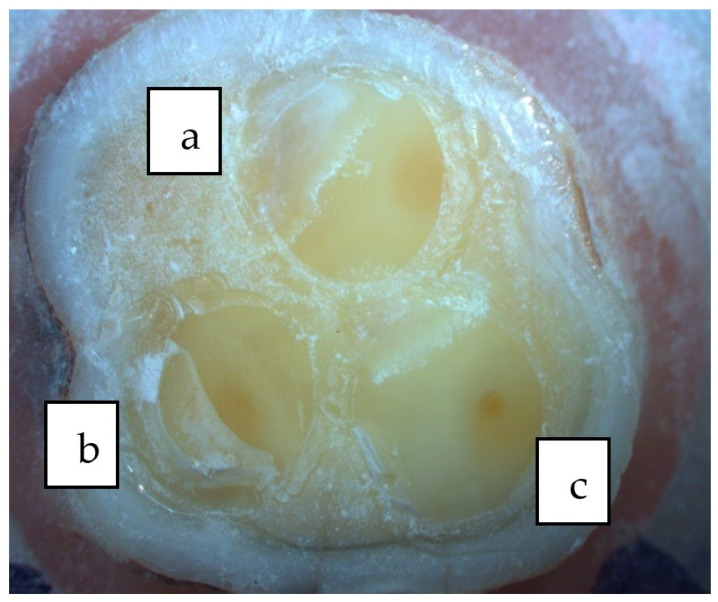
(a) Cohesive fracture, (b) mixed fracture, and (c) adhesive fracture.

**Figure 11:**
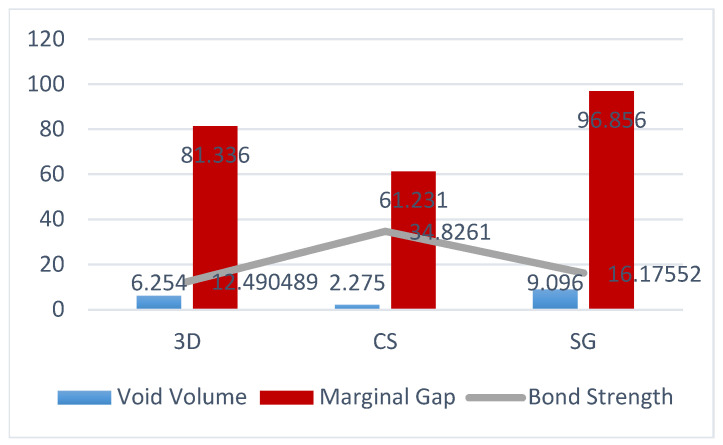
Comparative evaluation of bond strength and void volume percentages of 3D, CS, and SG groups.

The most prevalent fracture type was mixed fracture, accounting for 60% of the fractures (Figure 10). A comparative examination of marginal gap, average void volume, and bond strength was performed for all groups, and a significant positive correlation was found between the groups (Figure 11).

## 4. Discussion

Before a new material and production method can be routinely applied, important factors need to be investigated. In this study, the marginal fit and bond strength of indirect restorations manufactured with 3D printing were investigated.

One of the crucial factors influencing the quality and longevity of indirect restorations is the marginal fit. A poor marginal fit leads to the dissolution of adhesive cement and microleakage [19]. In turn, microleakage can result in postoperative sensitivity, secondary caries, and a weakening of mechanical properties [20]. Apart from the gaps that reduce marginal fit, it is known that the voids formed within the material also affect the prognosis of the restorations. Additionally, the presence of voids in resin composites or polymer-based dental materials has a significant impact on their numerous properties. Some of the main shortcomings of voids in mechanical properties include decreases in compressive and shear strength, fatigue resistance, and fracture toughness. To detect and monitor these voids within materials, a wide variety of non-destructive testing (NDT) methods can be used [21]. Among these NDT methods, some procedures are meant to be used for small specimens, such as radiographic testing (RT) and microcomputed tomography (micro-CT), while others are more suitable for larger samples. 

Microcomputed tomography, which has been increasingly used in recent years to evaluate marginal fit, is the most objective and clear method for evaluating the fit of restorations. The micro-CT method is considered a more noninvasive method than other methods, and it provides detailed information for evaluating the fit between the restoration and the tooth without damaging the specimen owing to the use of a three-dimensional high-resolution imaging system [20,22]. In addition, micro-CT has some advantages over some NDT methods, such as ultrasonic testing (UT) and infrared thermography (IRT) [21]. The UT method, which does not provide highly accurate information on void morphology and dimensions as micro-CT and IRT methods that have a resolution usually in the range of millimeters, is not efficient for tiny specimens, such as dental tissues and restorations.

In our study, because of the above-mentioned reasons, the micro-CT method was employed to measure marginal fit. According to the results of our study, the average measured marginal gap values were 81.33 µm for the 3D group, 61.23 µm for the CS group, and 96.85 µm for the SG group. There were no significant differences between the 3D group and the CS and SG groups in terms of marginal fit values. However, the marginal fit of the CS group was significantly better than that of the SG group. In studies examining marginal fit, differences in measurement techniques, reference points, and terms used can lead to variations in the results. In the most cited in vitro study involving 1000 crowns, a marginal gap of less than 120 μm was reported to be linked to a high success rate [23,24]. Regarding onlay restorations manufactured with 3D and other methods, we affirm that the measured marginal fit values were within clinically acceptable levels. The first initial hypothesis of our study posited that the marginal fit of indirect restorations produced with three-dimensional printers would be acceptable, and that there would be no significant difference compared with that of other methods. The results of our study have confirmed this hypothesis.

According to studies comparing the accuracy and marginal fit of onlay restorations produced by 3D printing and milling, there are no significant differences between the two methods [4,25,26,27]. In our study, no significant differences in the marginal fits of the 3D and CS groups could be observed, which aligns with the results of these published studies. In another study, it was reported that the marginal fit of 3D-printed onlays was significantly lower than that of onlays produced by the subtractive technique [28]. In this study, the onlay preparation covers only the mesio-buccal tubercle. Scholars have previously indicated that the marginal incongruence of onlay restorations tends to decrease with an increase in the number of cusps covered, and the best fit is achieved when all cusps are covered with restorative material [29]. In our study, reduction is performed on both lingual cusps, and the supporting cusps are covered with restorative materials. We believe that differences in cavity designs may have contributed to variations in marginal fit results between the two studies.

Another factor affecting marginal fit is the impression technique. Researchers comparing the impacts of traditional and digital impression methods on marginal fit have indicated that both methods meet clinically accepted standards. However, it has been determined that digital techniques provide better marginal fit than traditional techniques [19,30,31]. Silicone impression materials and model plaster are more prone to deformation and are at greater risk of being affected by practitioner errors during procedures. Additionally, practitioner skill and experience play crucial roles in determining the marginal fit of indirect composite resins applied to plaster models using traditional methods. Previous studies support the notion that the greater average marginal fit in the SG group can be attributed to the use of traditional methods.

In addition, the groups were evaluated based on their marginal fits in the cervical, middle, and occlusal regions. In the SG group, the region with the highest marginal gap was the middle region. In studies assessing the marginal fit of inlay/onlay restorations, the areas where the marginal gap was most frequently observed were concentrated at the gingival border, pulp, and axial wall regions [32]. The area referred to as the middle region in our study included the axial and pulpal wall boundaries. In the SG group, the increased concentration of gaps in this region was consistent with the literature.

In the 3D group, the marginal gap was significantly greater in the occlusal region. Due to the surface stepping phenomenon in 3D printing, occlusal surfaces or large curved surfaces are more prone to errors than vertical surfaces [33]. A study on the clinical application of 3D-printed restorations showed that the occlusal surface appeared different from the original surface after printing. Reportedly, the morphology needed to be corrected [26,34]. In a study evaluating the marginal fit of restorations produced by 3D printing and milling, the highest gap values could be found in the occlusal regions in restorations produced by 3D printing [35]. The direction of upside-down pressure from the gingiva to the occlusal space in 3D printing may be considered effective in these patients. Our findings are consistent with these referenced studies. Another important parameter that plays a role in marginal fit is the restoration material. VarseoSmile Crown Plus (VSCP) was used as the 3D printing material in our study. This material is approved for use in single-tooth restorations, such as full crowns, inlays, and onlays [36]. The chemical content is as follows. 4,4-Isopropylidiphenol is the ethoxylated and esterification product of 2-methyl-prop-2-enoic acid. The matrix contains 50–75% Bis-EMA by weight. Additionally, this material contains silane-coated dental glass particle fillers, methyl benzoyl-formate, and diphenyl (2,4,6-trimethylbenzoyl) phosphine oxide. Inorganic fillers with a particle size of 0.7 μm constitute 30% to 50% of the content by mass [37]. In a recent study by Shivaraman, marginal fit was carried out by producing crowns from zirconia materials via 3D printing, VSCP, and milling. Notably, 3D-printed VSCP crowns have similar edge compatibility to milled zirconium crowns after calculating the geometric volume differences with three-dimensional virtual software (3Shape Dental System version 2021) [37].

Although there have been numerous studies investigating the marginal fits resulting from layered manufacturing, there are insufficient studies exploring the potential for void formation during production and the resin material suitable for this technique. The scope of our study involves examining the layering style inherent in DLP 3D printers to assess the volume of voids within the restoration material and to compare it with clinically established methods.

In line with this objective, void volumes within sections corresponding to the occlusal, middle-third, and cervical regions obtained from microtomography scans were calculated using software. An analysis of the average void proportions within the restoration material for the 3D, CS, and SG groups indicated that the SG group exhibited the highest mean void-to-volume ratio, followed by the 3D group. The lowest void-to-volume ratio was observed in the CS group. A statistically significant difference could be identified between the SG and CS groups. However, the relationships between the 3D group and both the CS and SG groups were not significant.

In the SG group, where the average void volume was the highest, the layering technique could be applied to indirect resin composites on gypsum models, followed by the polymerization process. It is known that voids in composite materials created using the layering technique are most commonly formed at the junction points of layers. While the layering technique minimizes the effects of polymerization shrinkage, there is a risk of air void formation between composite layers. This situation can negatively impact the long-term success of restorations [38]. Various factors, such as the technique used during application, material properties, equipment, and practitioner skills, can influence the formation of voids within the composite material. McCabe and Ogden conducted a study in which they examined the voids in restorations produced using bulk-fill, incremental, and pressure-assisted composite placement techniques for the fabrication of inlay restorations. The results of this study indicated that composite application with pressure generated the fewest voids [38].

The layering technique employed in the 3D printing process for indirect composite resins is fundamentally similar to the additive manufacturing principle. During layered manufacturing, the bonds between layers are weaker than those within each layer. This phenomenon can be attributed to the accumulated residual stresses and pores that form during UV polymerization and material shrinkage [39]. The formation of voids in the 3D group is believed to have involved various factors, in addition to the layered manufacturing method, both before and during the printing process.

Resins used for DLP/SLA printing are typically supplied by manufacturers in bottles. During the printing process, resin composites are mixed or stirred according to the manufacturer’s instructions to ensure the homogeneity of the material; subsequently, this mixture is poured into the reservoir of the DLP/SLA printer. Since this process is conducted under atmospheric pressure, it can lead to the formation of voids [40]. Additionally, during production using the DLP/SLA technique, voids may form during the printing process. After the polymerization of a layer is complete, the platform separates from the base of the reservoir, and this separation process may introduce air into the resin by creating negative pressure. Reportedly, the viscosity of resin influences void formation, and increased resin viscosity is associated with reduced void formation. In an in vitro study conducted by Keßler et al., the mechanical properties of temporary 3D printing materials were examined. Fractographic analysis indicated that voids within the material were primarily located between two adjacent layers. In this study, we emphasized that voids in a product produced via 3D printing can adversely affect its mechanical strength. Moreover, mixing the resin under vacuum conditions before pouring it into the reservoir and using a resin with a low viscosity are recommended to overcome void formation [40].

The group with the lowest void volume within the restoration material could be identified as the CS group. The polymerization of nanohybrid blocks is conducted at high temperatures and pressures. These materials, containing glass particle fillers infiltrated into the resin matrix, are transformed into restorations through subtractive manufacturing. The void-to-volume ratios within these materials, subjected to high polymerization in a single stage, are considerably lower than those in other groups. Void formation between layers, observed in additive manufacturing, is not observed in subtractive manufacturing.

The bond strength between a fixed indirect restoration and adhesive is critically important for clinical longevity and stability. It is believed that shear stresses contribute significantly to the degradation of adhesive bonds and the occurrence of bonding failures in restorative materials in vivo. To predict clinical performance, shear bond strength testing is recommended, given that it is considered one of the most reliable testing methods. The test is particularly valuable due to its applicability. The shear bond strength test conducted with cross-sectional areas of 3 mm^2^ or less is referred to as microshear testing. Microshear tests are more reliable than macro shear tests [41].

The CS group exhibited a greater bond strength than the 3D and SG groups. Our second hypothesis posits that the bond strengths of indirect restorations produced with three-dimensional printers were acceptable and did not significantly differ from those of indirect restorations produced with other methods. However, based on the results of the bond strength tests, this hypothesis is rejected.

There have been few studies involving investigations of the bond strength of 3D printing resins. However, when comparing our bond strength test findings with those of a similar study in the literature, we observe that the results support each other. According to a study conducted by Dönmez et al., regardless of the bonded surface, resin nanoceramic blocks produced by subtractive methods exhibit greater bond strengths than additively produced 3D-printed hybrid composite resins [42]. Resin matrix ceramic blocks produced using a controlled and optimized polymerization process exhibit better bonding performance and demonstrate dentin-like behaviors. Our results support the findings of this study. A significant decrease in bond strength after thermal cycling could be observed in the CS group [43,44,45]. The results of studies examining the effects of thermal cycling on the bond strength of resin matrix ceramics are consistent with our findings. This phenomenon can be attributed to the diffusion of water into the matrix, followed by the leakage of unreacted monomers, expansion of the matrix, and weakening of the polymer structure of the resin. Celik et al. emphasized that thermal cycling reduces the bond strengths of resin-matrix ceramic materials [46].

In the SG group, there were no statistically significant differences between the bond strengths before and after thermal cycling. However, a slight increase in the average bond strength could be observed after thermal cycling. Lankes et al. investigated the effects of thermal cycling on the shear bond strength and tensile bond strength of 3D-printed resin bonded to composite resin and reported that thermal cycling positively affected bond strength [47]. Additionally, numerous scholars have indicated that applying additional heat to light-polymerized composite resins increases the degree of conversion, thereby reducing the residual monomer amount [48,49,50]. The observed increase in the SG can be explained by the promotion of the post-polymerization process at high temperatures. The average bond strengths of the 3D group were lower than those of the CS and SG groups. Notably, for objects produced by 3D printing, the likelihood of residual monomer retention is greater than that for subtractive resin matrix ceramics and composite resins [36]. As the number of monomers constituting the organic matrix decreases, the number of residual monomers decreases [51].

Following 3D printing, the final product is subjected to an ethanol bath aimed at reducing the residual monomer content, after which it undergoes polymerization within a light device. Despite this post-polymerization process, the lower average bond strengths of the 3D printing group can be attributed to differences in the matrix monomer content and material proportion.

According to the findings of our study, the shear bond strength of 3D-printed resin after thermal cycling is 11 MPa on average. There is no standardized format for interpreting the bonding properties of the data. Each study is conducted with different devices and operators. Factors such as material storage conditions and surface treatments contributed to the differences in the results. Therefore, it can be argued that data related to bond strength can be compared only within the same study groups [52].

The bond strength of VarseoSmile Crown Plus material to dentin is investigated in a study, and most of the connection failures are identified as mixed-type failures [42]. In a study examining the impact of shear bond strength, all the samples bonded with the 3D-printed resin Bis-GMA exhibited a mixed fracture tendency [53]. The results we obtained regarding the fracture tendencies of the 3D and SG groups are consistent with those of previous studies. A linear relationship has been reported between cohesive fracture tendency and the ability to withstand occlusal loads.

In additive manufacturing, one of the most fundamental factors affecting the success of the final restoration is the speed at which the system prints the layers, the selected solubility level, and the curing time determined to polymerize each layer [54]. Although there have been studies in the literature examining the production of dental restorations with different systems, there have been very few studies examining the effect of changes made in the production speed or parameters [27,55]. In a study conducted by Jockush and Ozcan, they stated that there are many factors affecting the production speed and reported that the most important parameters to obtain the best results from the polymer-based resin used in the production of dental restorations are viscosity and high speed in the polymerization process [54]. Factors such as the selected resolution level, the thickness of the layers, the curing time for each layer, and the movement distance of the table along the z axis are effective in determining the production speed. For this reason, different parameters can be tested to examine whether the final product to be obtained is mechanically and physically sufficient and compatible. However, since 3D printer technology is still a new field in dentistry and the systems are limited in number, the studies generally do not exceed the manufacturer’s optimum production instructions. During the examination of the marginal adaptation phase in our study, it was determined that the restorations produced by additive and subtractive methods showed the best adaptation. The reason for this is that the designs of the restorations were made with the CAD system and then their production was prepared in accordance with the manufacturer’s instructions. In our study, during the examination of the marginal adaptation phase, it was determined that the restorations produced by additive and subtractive methods showed the best adaptation. The reason for this is that the designs of the restorations were made with the CAD system and then their production was prepared in accordance with the manufacturer’s instructions.

## 5. Conclusions

In this study, 3D printing technology is found to be a promising option for indirect restorations. Although marginal fit evaluation proves the fact that both additive and subtractive methods are equally adequate and effective in manufacturing dental restorations, minimal errors and deviations may be encountered in the adaptation of restorations depending on the selected CAD design system and the clinical experience of the physician. On the other hand, mechanical properties, such as bond strength, microhardness, and wear resistance, are directly affected by the properties of the polymer-based resin used, the parameters in the production phase, and the cleaning and re-curing processes in the post-production phase. Even the thermal cycle results showed that the contact of polymer-based resins with heat during or after the curing phase increases the physical strength of these materials. For these reasons, further research and clinical studies are needed to confirm this potential. The technical specifications of 3D printing, printing angle, layer thickness, curing procedures during and after printing, development of components in 3D printing resin, and surface preparation processes are crucial areas that need to be addressed to enhance the success of restorations produced with 3D printing.

## Figures and Tables

**Table 1:** Evaluation of marginal fits in manufacturing methods.

Groups	N	Mean (µm)	*p*-Value
3D	60	81,336	
CS	60	61,231	0.007
SG	60	96,856	

**Table 2:** Bond strength test results.

Groups	Pre-TC Bond Strength (MPa)	Post-TC Bond Strength (MPa)	*p*-Value
3D	12.49 ± 2.83	11.36 ± 5.41	0.586
CS	34.82 ± 6.40	26.87 ± 9.38	0.036
SG	16.17 ± 6.10	17.34 ± 4.31	0.710

## Data Availability

Data are contained within the article.
